# A fatal toxic shock-like syndrome post COVID-19 infection in a child

**DOI:** 10.1186/s13052-021-01070-z

**Published:** 2021-06-02

**Authors:** Houda Ajmi, Wissem Besghaier, Wafa Kallala, Abdelhalim Trabelsi, Saoussan Abroug

**Affiliations:** 1 Department of Pediatrics, Sahloul University Hospital, 4054 Sousse, Tunisia; 2 Department of Virology, Sahloul University Hospital, 4054 Sousse, Tunisia

**Keywords:** Toxic shock syndrome, Multi-system inflammatory syndrome, Coronavirus infection, Child, TSS: toxic shock syndrome

## Abstract

**Background:**

Children affected by Coronavirus disease 2019 (COVID-19) showed various manifestations. Some of them were severe cases presenting with multi-system inflammatory syndrome (MIS-C) causing multiple organ dysfunction.

**Case presentation:**

We report the case of a 12-year-old girl with recent COVID-19 infection who presented with persistent fever, abdominal pain and other symptoms that meet the definition of MIS-C. She had lymphopenia and a high level of inflammatory markers. She was admitted to pediatric intensive care unit since she rapidly developed refractory catecholamine-resistant shock with multiple organ failure. Echocardiography showed a small pericardial effusion with a normal ejection fraction (Ejection Fraction = 60%) and no valvular or coronary lesions. The child showed no signs of improvement even after receiving intravenous immunoglobulin, fresh frozen plasma, high doses of Vasopressors and corticosteroid. His outcome was fatal.

**Conclusion:**

Pediatric patients affected by the new COVID-19 related syndrome may show severe life-threatening conditions similar to Kawasaki disease shock syndrome. Hypotension in these patients results from heart failure and the decreased cardiac output. We report a new severe clinical feature of SARS-CoV-2 infection in children in whom hypotension was the result of refractory vasoplegia.

## Background

Coronavirus disease 2019 (COVID-19) is a pandemic caused by severe acute respiratory syndrome Coronavirus 2 (SARS-CoV-2) affecting individuals of all ages [1, 2]. It can lead to multi-system inflammatory syndrome in children (MIS-C), also known as pediatric inflammatory multi-system syndrome. Children with this syndrome often had severe symptoms resembling those of well-defined entities such as Kawasaki disease or macrophage activation syndrome [1, 3, 4]. Few of them developed refractory catecholamine-resistant shock along with multi-organ dysfunction mimicking toxic shock syndrome (TSS) [1, 3–5].

We report a young Tunisian girl recently infected with SARS-CoV-2 who presented features of MIS-C with multi-organ injury resembling to TSS.

## Case presentation

**Fig. 1:**
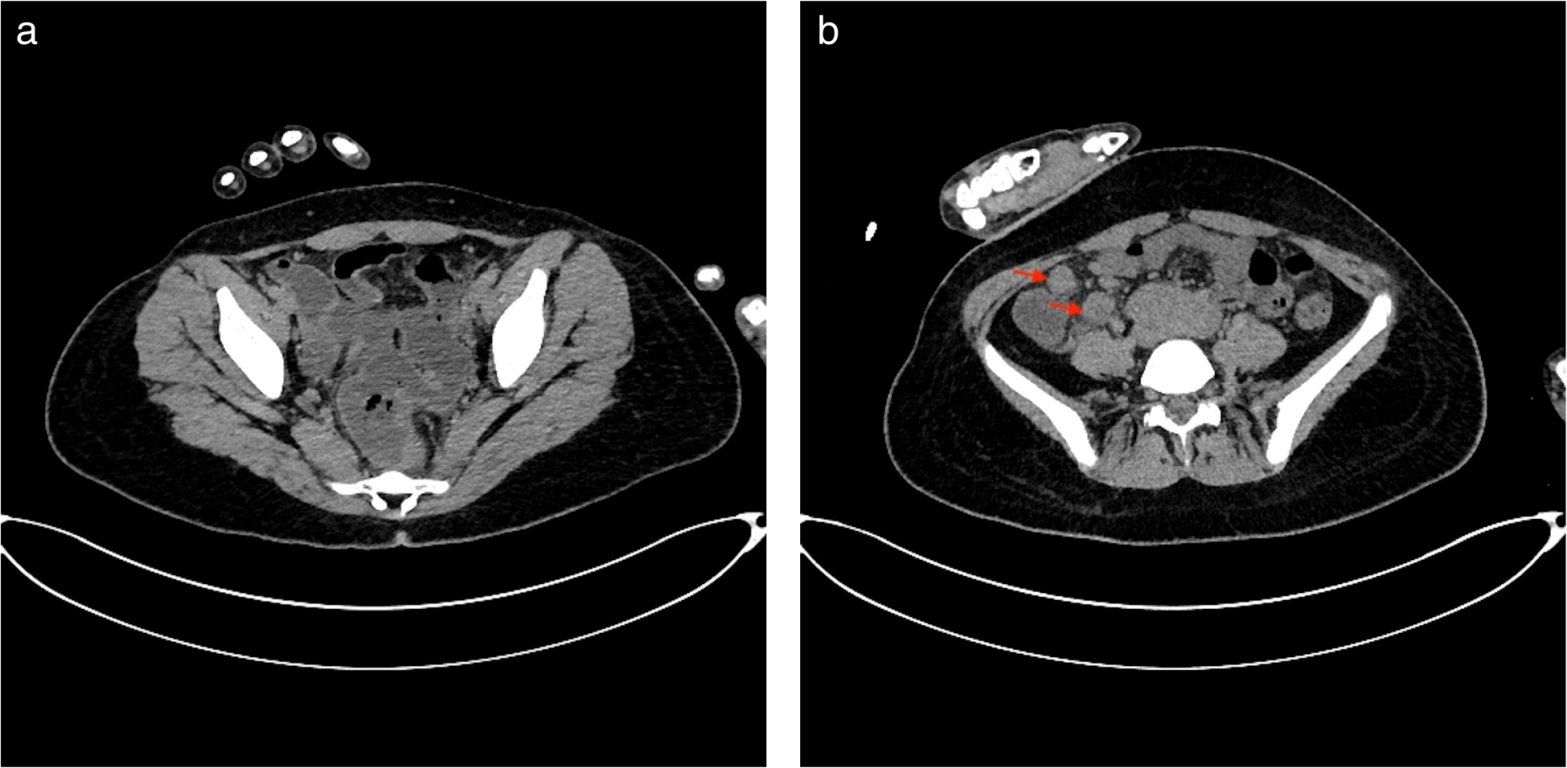
Abdominal computed tomography showing (**a**) slight thickening of the rectosigmoid colon, (**b**) multiple enlarged lymph nodes in the right lower quadrant

**Fig. 2:**
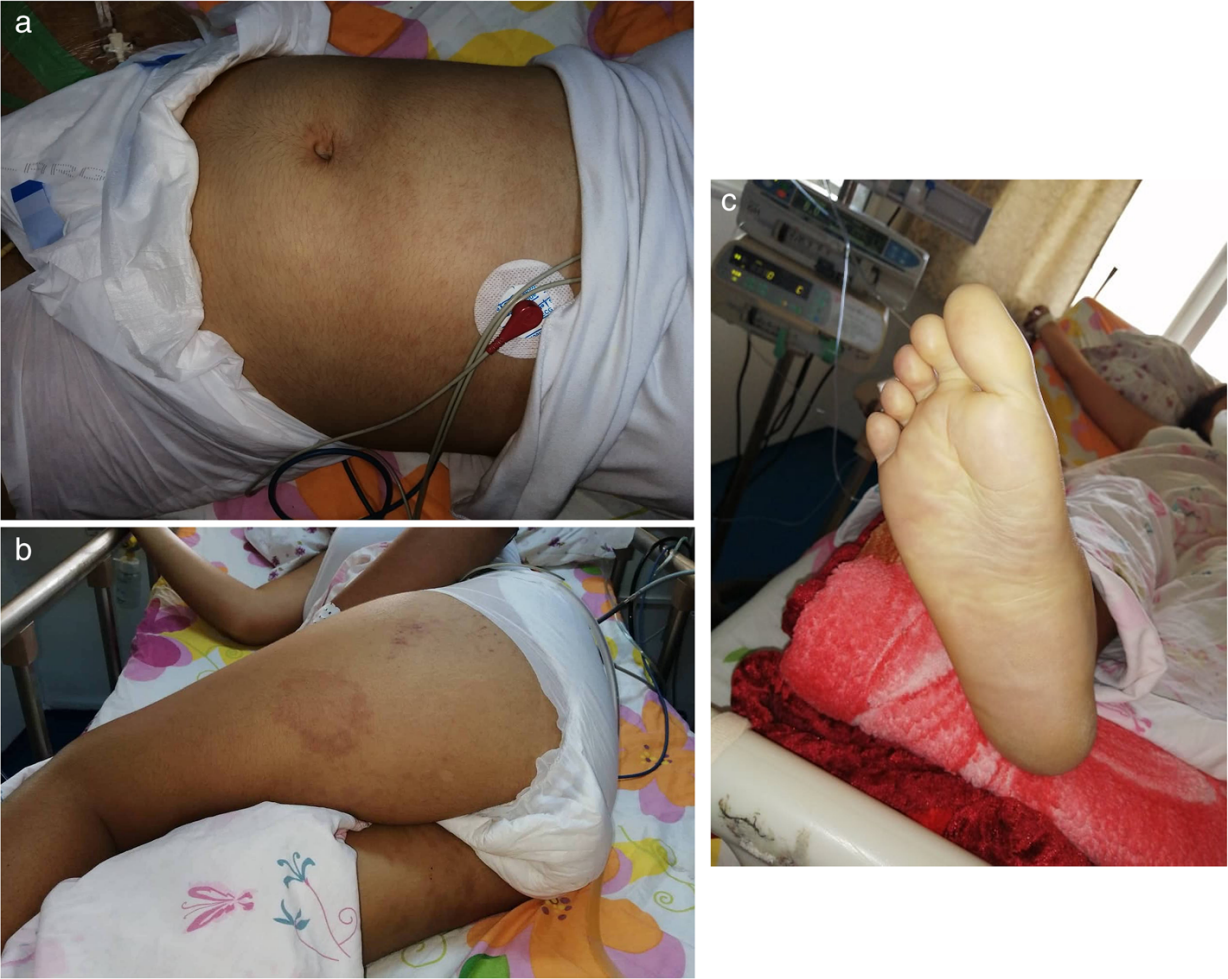
Maculopapular rash in abdomen (**a**), thighs (**b**) and feet soles (**c**)

A 12-year-old girl has been hospitalized with fever, headache, vomiting, and abdominal pain. She had a history of contact with COVID-19 patients and her test for SARS-CoV-2 by Reverse Transcription Polymerase Chain Reaction (RT-PCR) was positive 15 days prior to her admission. Physical examination showed fever, irritability, and bilateral submandibular adenopathy. On abdominal palpation, she had a diffuse tenderness, most pronounced over the right lower quadrant mimicking an acute surgical abdomen. Sonography and Computed Tomography Scan revealed pelvic ectopy of her right kidney. Laboratory tests showed lymphopenia (total leukocyte count = 16 × 10^9^/l; lymphocyte count = 0.9 × 109/l) with elevated inflammatory markers (C-reactive protein = 127 mg/l). Renal and liver functions were correct. Urinary cytobacteriological investigation showed the presence of leucocyte in urine (988/mm^3^). Therefore, urinary tract infection was initially suspected and the child was put on intravenous antibiotics (cefotaxime and gentamycin) for 3 days without any improvement. Urine culture and hemocultures come back later negatives. The girl had persistent fever, diarrhea, and severe abdominal pain. She also developed hypotension (76/31 mmHg) and tachycardia (150/min). She was transferred to our Pediatric Intensive Care Unit after fluid resuscitation and norepinephrine infusion. Broad-spectrum antibiotics were introduced including imipenem, vancomycin and amikacin. Laboratory exams revealed elevated C-reactive protein (359 mg/l), high level of transaminases (SGOT = 491 UI/l / SGPT = 184 UI/l), renal dysfunction (urea = 15 mmol/l; creatinine = 360 μmol/l), hyponatremia (129 mmol/l), and hypokalemia (2.6 mmol/l). Complete blood count showed lymphopenia (total leukocyte counts = 15.95 × 10^9^/l; lymphocyte count = 0.6 × 10^9^/l) and thrombocytopenia (platelet count = 110 × 10^9^/l). Blood gas analysis revealed metabolic acidosis with pH = 7.06; PaO2 = 40 mmHg; PaCO2 = 17 mmHg; HCO3- = 4.8 mmol/l and hyperlactatemia at 6.4 mmol/l. Computed Tomography Scan showed a few posterior pulmonary consolidation lesions associated with a small pleural and pericardial effusion, hepatomegaly, recto-sigmoiditis (Fig. 1a), mesenteric lymphadenopathy (Fig. 1b), pelvic ectopic right kidney, and a small pelvic peritoneal effusion, without any signs of appendicitis or peritonitis. Echocardiography demonstrated the existence of a small pericardial effusion with a normal ejection fraction (Ejection Fraction = 60%) and no valvular or coronary lesions. A second test for SARS-CoV-2 by RT-PCR was negative as well as hemocultures and urine culture. Dobutamine and hydrocortisone were added because of persistent hypotension despite multiple fluid resuscitation and increasing norepinephrine infusion. Clinically, multiple mucocutaneous lesions appeared including maculopapular rash (Fig. 2a, b, c), and subconjunctival hemorrhage. A worsened hypoxemia was noted along with neurological troubles, leading to respiratory failure and requiring intubation and assisted-ventilation. Blood test showed decreased count of platelets at 56 × 10^9^/l and prothrombin time at 33% (Table 1). Platelet and Frozen Fresh Plasma transfusion were indicated. It was a refractory shock with multiple organ failure. The diagnosis of MIS-C with features of TSS was suspected. Therefore, the child was treated with intravenous immunoglobulin (IVIG) (1 g/kg/day for two days). Despite of cardio-pulmonary resuscitation, the patient had a fatal outcome.

**Table 1:** Evolution of biological data of the patient during hospitalization in Pediatric Intensive Care Unit

	H1	H12	H20	H26	H36	H42	H48
**Leukocytes (**×10^9^/l)	15.950	20.920			32.500		
**Hemoglobin** (g/dl)	11.1	11.5			8.9		
**Platelets** (×10^9^/l)	111	68			56		
**PT**	73%				33%		
**CRP** (mg/l)	359				219		
**Na +** (mmol/l)	129	131	134	137	140	142	136
**K+** (mmol/l)	2.6	3.9	5.2	5.1	4.6	4.8	6
**Urea** (mmol/l)	15.7	18	18.4	17.5	17.3		
**Creatinine** (μmol/l)	360	350	356	304	256		
**AST/ALT** (UI/l)	47/31				491 / 184		
**CPK/LDH** (UI/l)		240 / 422					864 / 1342
**pH**		7.06	7.01	7.1	7.21	7.34	7.00
**PaO**_**2**_ (mmHg)		40	50	82	95	134	39
**PaCO**_**2**_ (mmHg)		17	13	13	13	18	54
**HCO**_**3**_^**−**^ (mmol/l)		4.8	3.3	4	5.2	9.7	13.3
**SatO**_**2**_		50%	61%	91%	96%	99%	44%
**Lactates** (mmol/l)		6.4	8.9	11.4	11.7	12.9	11.1

ALT: alanine transaminase; AST: aspartate transaminase; CPK: creatine phosphokinase; LDH: lactate deshydrogenase; PT: prothrombin time

## Discussion and conclusions

The clinical presentation of our patient associating fever, hypotension, diffuse skin rash, and multisystem organ dysfunction was initially suggestive of septic shock complicated by TSS. After having had negative results from the bacteriological analysis and having considered the COVID 19 pandemic, however, we have raised concerns for serious syndromes caused by this virus such as MIS-C. The patient fulfilled MIS-C criteria and showed overlapping features with Kawasaki disease shock syndrome (KDSS) and toxic shock syndrome. We finally retained the diagnosis of MIS-C with features of TSS secondary to COVID19 considering the normal heart function and the absence of coronary lesions. TSS is a severe syndrome secondary to the uncontrolled activation of the immune system by “superantigens”, proteins that stimulate T cells, leading to a massive production and release of cytokines [3, 6]. Bacterial species, such as *Staphylococcus aureus* and *Streptococcus pyogenes*, are well-known to produce exotoxins that can function as superantigens. Interestingly, some viruses including SARS-CoV-1 can also act as superantigens [3, 6]. As for SARS-CoV-2 this “superantigen” role has not yet been proved. However, the hyperinflammatory state and cytokine storm noticed in patients, who are infected by SARS-CoV-2 may suggest the existence of a similar immunological phenomenon to the one described in TSS [1, 3, 7–9]. As in the majority of patients having MIS-C, patient presenting features of TSS due to COVID19, usually had a negative RT-PCR test, while the serologic testing for SARS-CoV-2 reported positive in many cases [1, 3, 7–9]. In our case, 2 weeks after being tested positive for SARS-CoV-2 by RT-PCR, the patient developed symptoms and biological abnormalities fulfilling the definition of MIS-C. Her negative second test by RT-PCR elucidate the post-infectious MIS-C’s pathogenesis’s theory. Typical treatment for TSS includes fluid resuscitation and pressor support [10]. The majority of reported children responded well to those treatments with the resolution of hypotension after volume resuscitation and only a few cases required the use of inotropes. The use of IVIG and corticosteroids in TSS remains controversial. Chen et al. [11] reported 62 children with TSS, 24 of them received corticosteroid. In this study, the residual morbidity was higher in patients who received IVIG or steroids. The authors explained that these results may be due to the fact that these treatments were given to more seriously ill patients. However, others authors reported that the use of IVIG has improved the outcome of patients with TSS [12]. In patients with MIS-C, the optimal therapy remains unknown [13]. Empirical treatment with IVIG alone or associated with corticosteroids has been received by many children with MIS-C based on Kawasaki disease guidelines [13–16]. Previously, In patients with MIS-C, corticosteroid has been provided as a second-line therapy for children who remain unwell 24 h after infusion of IVIG or in those with severe symptoms [3, 17, 18]. Actually, steroids such as methylprednisolone may not be used as rescue therapy but rather as a first line therapy for MIS-C. Ouldali et al. has compared IVIG plus methylprednisolone vs IVIG alone as initial therapy in MIS-C through a retrospective cohort study of 106 patients with confirmed MIS-C [13]. They showed that only 9% of children treated with IVIG and methylprednisolone did not respond to treatment vs 51% in the IVIG alone group. In this study, IVIG and methylprednisolone therapy compared to IVIG alone was also significantly associated with lower risk of use of second-line therapy, hemodynamic support, acute left ventricular dysfunction occurring after initial therapy, and duration of stay in the pediatric intensive care unit. Methylprednisolone was given to patients at a posology of 1.6 mg/kg to 2 mg/kg/d for 5 days or as a bolus of 15 to 30 mg/kg/d for 3 days. In our patient there was no signs of improvement after receiving IVIG. The child received only hydrocortisone, for the refractory shock to deal with an eventual acute adrenal failure. He didn’t receive methylprednisolone. And the outcome was rapidly fatal. High doses of methylprednisolone are useful to have a powerful anti-inflammatory effect, to fight the inflammatory storm. Jonat et al. [19] have developed a protocol for the evaluation, management, and follow-up of patients with MIS-C. This protocol states that all patients fulfilling MIS-C criteria should receive IVIG plus corticosteroids. The recommended dose of steroids is variable depending of the severity of the clinical presentation. Severe cases should receive methylprednisolone 20–30 mg/kg/d for 1–3 days (maximum 1 g/d), followed by 2 mg/kg/d (maximum 60 mg/d). In these severe cases, steroids should be tapered slowly to avoid rebound illness. Refractory patients to pulse glucocorticoids should be treated by Anakinra [19]. Other biologic immunomodulators may be considered in severe cases with no response to anakinra. Infliximab and tocilizumab, used in other hyper-inflammatory syndromes as biologic immunomodulators have been tried to modulate the dysregulated hyper-inflammation apparent in MIS-C with promised result in adults [20, 21]. However, there is limited evidence to suggest these therapies in children.

## Data Availability

The authors did not use any database, software, or tools for the writing of this manuscript.

## Abbreviations

COVID-19: Coronavirus disease 2019
IVIG: intravenous immunoglobulin
KDSS: Kawasaki Disease shock syndrome
MIS-C: Multi-system inflammatory syndrome in children
RT-PCR: Reverse Transcription Polymerase Chain Reaction
SARS-CoV: Severe acute respiratory syndrome coronavirus
